# Neck Cutting Behavior: Paraphilia or Suicide Attempt? A Case Report of Self-harm in the Context of Drug Abuse and Depression

**DOI:** 10.7759/cureus.3332

**Published:** 2018-09-18

**Authors:** Nathan Sherman

**Affiliations:** 1 Medicine, University of Arizona School of Medicine, Tucson, USA

**Keywords:** cutting behavior, neck, depression, paraphilia, bipolar disorder, drug abuse, suicidal ideation, suicidality

## Abstract

The border between self-harm and suicidal behaviors is not always clear. Self-mutilation is a common finding in mood and personality disorders, and cutting of the extremities is more common than cutting of the neck. The case put forth regards a young adult male with a past history of depression and drug abuse who presented to the emergency department with superficial lacerations on his left arm and bilaterally on his neck with a large abscess in his right forearm. The patient reported the cuts on his arm to be from testing the sharpness of a kitchen knife and the cuts on his neck to be the result of sexual activity between him and his girlfriend. Collateral reports were inconsistent with the patient’s version of events, and he voluntarily chose to receive psychiatric evaluation. After undergoing hospital rehabilitation, the patient was discharged with the diagnosis of bipolar disorder unspecified due to the findings of chronic mood instability without conclusive evidence of mania or hypomania. The etiology of his neck cutting behavior remained unexplained.

## Introduction

Individuals who have self-harmed, as in the case of our current patient, who cut his left wrist and his neck bilaterally, have a suicide risk that is 50 times greater than the suicide risk in the general population [1]. While cutting behavior has a higher incidence in individuals with mood or personality disorders [2], those who cut in areas other than the arms have a higher risk of subsequent suicide than those that cut only on the wrists or that poison themselves [1]. Thus, neck cutting behavior represents a clinical finding for which the prognosis is unclear.

Self-harm remains a stigmatized condition in which both socioeconomic and cultural factors play a role. Low socioeconomic status is an established risk factor for self-harm [3], and cultural perceptions regarding behavior, self-harm, and suicide can vary widely. Furthermore, there are ethnic differences in regard to the prevalence of self-harm. For example, Asian males are least likely to self-harm, while African American females are most likely to self-harm, and cultural influence is likely the predominant reason for this particular trend [4].

There is an established relationship between self-harm and suicide. In 2002, it was estimated that there was approximately one suicide for every 12-15 hospitalizations related to self-harm [5]. Since that time, suicide rates have continued to rise [6], particularly in nonmetropolitan and rural communities [7]. The strongest risk factor for suicide is preceding nonfatal self-harm, but male gender and more urgent medical care at the time of initial self-harm are also predictors of suicide after nonfatal self-harm [8]. Additional risk factors include drug and alcohol abuse or dependence, personality disorder, hopelessness, schizophrenia, and living alone [9]. Both suicide and self-harm are temporally correlated [10], but more than two thirds of suicides occur in individuals who have not displayed self-injurious thoughts or behaviors [11].

Despite their clinical correlation, self-harm and suicide are separate entities. While suicide is a result of self-harm, self-harm does not necessarily indicate a desire to commit suicide. To clarify this distinction, the diagnosis of nonsuicidal self-injury disorder (NSSID) is now included in the Diagnostic and Statistical Manual of Mental Disorders (DSM-5). Recently, however, it has been identified that suicidal ideation may occur before the initial engagement in self-harm, indicating a simultaneous process as opposed to a linear progression from self-harm to suicide attempt [12].

## Case presentation

The case presented here is one of diagnostic value and provides an unusual example of self-injurious behavior for which the suicidal intention is unclear. The patient is a male in his early 20s with a past medical history pertinent for complex partial seizure disorder, cocaine and intravenous heroin abuse for the last two years, opiate dependence, and depression who presented to the psychiatric emergency department under petition for suicidal ideation and self-injurious behavior involving multiple, superficial, bilateral lacerations on his neck, linear lacerations on his left forearm, and a swollen right forearm. The patient reported being unaware of the petition and that he was under the impression that he was there to “arrange for counseling.”

The patient reported a history of depression for multiple years diagnosed by his primary provider but had never received treatment for his condition and had no previous psychiatric hospitalizations. The patient had never suffered episodes of psychosis nor received assessment for personality disorders, and he had no known family history of psychiatric illnesses or attempted suicides. He was unemployed at the time of his admission and left college after his sophomore year but reported interests in business, culinary school, and now medicine as career choices.

He denied any suicidal or homicidal ideation and reported that the linear lacerations on his bilateral neck were “no big deal” because “my girlfriend and I just like to get K-I-N-K-Y.” When prompted more as to what that meant, he said in a rather boisterous way that he was embarrassed by the behavior but that his girlfriend would cut his neck with a sharp knife and lick the blood before having intercourse. He reported that this was not the first time they had done this together or the first time he had engaged in cutting behavior, but that the intensity of these encounters was increasing and that his cutting behaviors had never resulted in visible scars prior to this episode. He explained the lacerations on his left forearm, however, as an attempt to test the sharpness of the knives that he used working in a restaurant kitchen because, “That’s how you know if the knife is really sharp.” The patient demonstrated significant hesitation when asked if we could contact his girlfriend to verify the collateral information.

Notably, he also presented with a swollen right forearm without significant redness or warmth to touch that had gradually increased in pain over the previous few days. Computed tomography (CT) imaging of the right forearm identified a 7.6 cm multiloculated abscess and myositis involving the flexor musculature. His urine toxicology was positive for benzodiazepines, marijuana, and opioids. He reported most recently using intravenous heroin in that arm about two weeks prior and claimed that he was currently taking suboxone and clonazepam for drug rehabilitation and doxycycline for the suspected infection in his arm. He reported he had been using heroin for two years.

The patient’s mental status exam on presentation was notable for friendly and cooperative behavior, speech of rapid rate and volume, euphoric mood with expansive affect, and tangential thought processes but normal thought content without auditory or visual hallucinations or suicidal or homicidal ideation. He was oriented to person, place, and time, but not situation. His fund of knowledge and judgment were fair, but his insight was poor. He did not suffer from any obvious delusions.

The patient was admitted to the inpatient service for drainage of his abscess, at which time he voluntarily agreed to psychiatric hospitalization. It was discovered through collateral sources that the patient had on numerous occasions been encouraged to receive drug rehabilitation therapy for heroin abuse, but no psychiatric intervention had yet been attempted. According to the patient’s girlfriend, the patient’s behavior had been more erratic the few weeks prior. Collateral sources obtained text messages between the patient and his girlfriend that substantiate the notion that the patient was attempting to hurt and possibly kill himself with his self-inflicted neck lacerations. These same sources described the patient’s behavior as “manipulative,” saying that he could portray himself as a kind and ordinary individual when he wanted to do so, despite frequent verbally abusive outbursts to others, including his parents. The patient reportedly exhibited frequent episodes of spontaneous tearfulness along with mood swings, depression, racing thoughts, and elevated mood for weeks at a time. This had occurred commonly over the last several years, but it was unclear to the patient whether the onset of drug use preceded or was concurrent with these episodes. Based on collateral reports, the patient had a long history of risky and impulsive behaviors that predated the patient’s first reported use of heroin. Furthermore, the patient had been mandated to undergo court ordered drug rehabilitation treatment two years ago after two arrests for possession of heroin, but he never went to appointments.

## Discussion

One of the predicaments that the treatment team faced with the patient in question is whether to believe his statements regarding his wrist cutting behavior and the sexual nature of the lacerations on his neck. The patient’s explanation for his wrist cutting as testing the sharpness of a kitchen knife appears far-fetched and is unlikely to be true. While cutting on the extremities is well documented, and in this patient’s case a previous occurrence, superficial cutting of the neck seemed to represent an entirely different situation stemming from a potentially unique etiology. Were the cuts on his neck the result of “normal” sexual behavior, sexual deviancy, cutting behavior, or suicidality? To the patient, his neck cutting behavior was purported to be the result of “normal” sexual behavior, but to many, it would qualify as sexual deviancy. Collateral sources, however, interpreted the neck cutting behavior as representative of suicidality given previous experiences with the patient.

A sexual act is deemed a deviant behavior or paraphilia if it falls outside of the range of what is considered a “normal” sexual interest or if it infringes upon the rights of others. Depiction of certain sexual acts as paraphilias has fallen out of favor, perhaps due to societal changes or because of the distaste felt by many after homosexuality had historically been classified as a paraphilia in previous DSM editions. If this patient’s neck cutting behavior was indeed sexually motivated, then it would fall under the realm of masochism in the paraphilia classification, and he may benefit from further evaluation and psychotherapy. However, given the evidence obtained from text messages and the way in which the patient so openly acknowledged and boasted about his sexually-motivated reasons for his self-injurious behaviors, the treatment team felt that the patient’s neck cutting was not indicative of a masochistic paraphilia but instead more likely associated with cutting behavior and/or an undiagnosed mood or personality disorder.

It is important to differentiate cutting behavior from suicidality in the case of this patient. Suicide by cutting and/or stabbing is not as common as suicide by other means, such as hanging, firearms, drowning, poisoning, or falling from a height, and involves the neck in only 1/3rd of cases [13,14]. A para-suicidal behavior can unintentionally result in mortality if extreme in nature or complicated by euphoria. To the treatment team, the superficiality and location of the cutting above the midline of the neck combined with his lack of desire to conceal the cuts and his dramatic presentation of his sexual exploits appeared to be more of an overt gesture of his self-asserted “masculinity” or a cry for attention rather than an actual suicide attempt. While neck cutting may be an obscure representation of cutting behavior, the clinical picture often indicates the seriousness of the attempt. If the patient, for instance, had attempted to cut his neck via a deadlier method, as has been documented in previous studies [13,15], the treatment of the patient would likely have been much more aggressive.

Young adult males who abuse heroin are a vulnerable group and engage in more risky injection practices than older male heroin abusers [16]. Given that the patient had an abscess formation at his injection site, the treatment team also considered if drug-induced psychosis led the patient to cut his own neck and arm. While the patient appeared forthcoming, his urine toxicology findings were incongruent with his reports, as he tested positive for opioids. He reported suboxone use, but suboxone is a synthetic opioid that provides a different metabolite profile than that tested for in the standard urine toxicology used at our institution. Despite evidence that the patient had recently used opioids, there was no way to verify the timeline of the patient’s drug use, making drug-induced psychosis a difficult diagnostic option given the sub-acute nature of the presentation.

Borderline personality disorder is another diagnosis that received consideration. This disorder is one often characterized by dramatic, self-harming, or para-suicidal behavior [2]. Cutting, however, is the fifth most common form of self-harm for those with the disorder [19]. The patient did not exhibit classic splitting behaviors and expressed stable social and familial relationships despite collateral accounts to the contrary that his few social relationships were strained and impacted by his drug abuse. While non-compliance with drug treatment could also be interpreted as behavior commonly observed in individuals suffering from borderline personality disorder, non-compliance with treatment is not specific to this disorder alone and could be indicative of substance abuse disorder, bipolar disorder, or others. For these reasons, the patient was not diagnosed with borderline personality disorder.

After 10 days of treatment, including surgical incision and drainage of his abscess, intravenous antibiotics, and psychiatric consultation, the patient was discharged to home. The treatment team concluded that while the patient was clearly suffering from mood instability and self-injurious behavior, he was not actively suicidal and did not meet criteria for bipolar disorder type I or II, so he instead left the hospital with the diagnosis of bipolar disorder unspecified. He did not receive the diagnosis of substance-induced bipolar disorder because the timeline of his drug abuse and historical mood instability was unclear. Although he came close on several occasions, at no point during the patient’s stay did he meet full criteria for the diagnosis of either hypomania or mania, and the duration of his symptoms led more to the determination that he was suffering from chronic mood disturbances. Bipolar disorder portends an increased risk of suicide compared to the general population [17], and given that early treatment of bipolar disorder through psychiatric and pharmacologic means has been shown to improve clinical outcomes [18], the patient was started on olanzapine for mood stabilization and also prescribed lamotrigine, which he had previously taken for treatment of seizures. He remained on his previous regimen of suboxone and clonazepam monitored by an outside agency. While the patient agreed to seek private psychiatric counseling after his discharge, the patient once again appeared to focus on his use of drugs as the primary need for treatment rather than acknowledging the abnormal nature of his wrist and neck cutting behaviors or his historical mood instabilities.

In hindsight, additional testing should have been performed on this patient to further delineate his condition prior to his discharge. The recently developed Clinician-Administered NSSI Disorder Index (CANDI) has demonstrated reliable and valid results to-date [20]. A Mood Disorder Questionnaire (MDQ) or Composite International Diagnostic Interview 3 (CIDI 3.0) should have been performed in order to determine the more likely bipolar disorder variant, type I or type II [17]. Greater effort should also have been taken to delineate the timeline of drug abuse and to encourage the patient to confront his underlying mood disturbances.

## Conclusions

Young individuals with comorbid medical conditions and drug abuse presenting for the first time for cutting behaviors versus suicide attempt represent a challenging diagnostic dilemma. The care of these patients should be approached methodically with the careful collection of collateral information and a healthy dose of both diagnostic comparison and analysis. Furthermore, neck cutting behavior requires special diagnostic consideration as it may represent a clinically severe variation of cutting behavior, suicidal ideation, or even sexually-motivated paraphilia.
